# Effect of the Chronic Use of Lithium Carbonate on Induced Tooth Movement in Wistar Rats

**DOI:** 10.1371/journal.pone.0160400

**Published:** 2016-08-03

**Authors:** Viviane da Silva Kagy, Luciana Trevisan Bittencourt Muniz, Arieli Carini Michels, Suelen Teixeira Luiz, Luciana Reis Azevedo Alanis, João Armando Brancher, Ana Maria Trindade Grégio, Sérgio Aparecido Ignácio, Elisa Souza Camargo, Maria Ângela Naval Machado, Aline Cristina Batista Rodrigues Johann

**Affiliations:** 1 Department of School of Health and Biosciences of Pontifícia Universidade Católica do Paraná, Curitiba, PR, Brazil; 2 Department of Stomatology, School of Dentistry, Universidade Federal do Paraná, Curitiba, PR, Brazil; Boston University Goldman School of Dental Medicine, UNITED STATES

## Abstract

Patients who seek dental treatment may have bipolar disorder, and lithium carbonate (LC) is the drug of choice used in the treatment of this disorder. Taking into consideration the controversial results found in the literature, and the possible influence of LC on induced tooth movement, the objective was to evaluate tooth movement induced in rats after administration of lithium carbonate. One hundred and ninety-two rats were divided into 3 groups. In the L group, the animals received daily 60mg/kg of LC, they were not subjected to orthodontic movement, and they were euthanized after 33, 37, 44 or 51 days. In the LM group, the LC was administered for 30 days and during the subsequent 3, 7, 14 and 21 days, corresponding to the period of induced tooth movement, and they received a spring that produced a 30cN force. In the SM group, saline solution was applied. Measurements were made of tooth displacement, the numbers of osteoclasts and serum lithium phosphate (PO4), alkaline phosphatase (ALP) and creatinine levels. The tooth displacement was lower in the LM group compared to the SM group at 44 days. A tendency toward reduction in the number of osteoclasts was observed in the LM group compared to the SM group at 44 days. The average lithium were higher in the L and LM groups compared to the SM group. The opposite was observed for the PO_4_ group. A higher value for the ALP was found in the L group. The average creatinine level was lower in the LM group. LC inhibited tooth movement for 14 days, possibly due to the reduction in the number of osteoclasts.

## Introduction

Bipolar Disorder (BD) is a mental disorder that causes impairments in the functions of daily life. The prevalence of BD in the general population is estimated to be 1%, and lithium carbonate (LC) is the drug of choice used in the treatment of this disorder [1]. Patients who seek dental treatment may have BD [2], an issue being addressed in the orthodontic literature [3], emphasizing the necessity for research in this area. This drug is also used in the treatment of other psychological disorders such as unipolar depression in adults [4], attention deficit in children [5], and hyperactivity and obsessive-compulsive disorders in adolescents [6].

Orthodontic treatment is based on the premise that a force is applied to a tooth and transmitted to the surrounding tissues, causing chemical, mechanical and cellular events[7,8]. The specific changes that occur in the bone tissue and at the root of a tooth, which has undergone orthodontic treatment, are bone resorption by osteoclasts on the pressure side and bone deposition on the tension side[8–10].

The measurement of induced tooth movement using a digital caliper [11], the biochemical analysis of blood [9,10,12], and histochemical staining using tartrate-resistant acid phosphatase (TRAP) [13,14] are useful methods for the evaluation of induced tooth movement.

The tissue changes produced by orthodontic force can be influenced by local factors related to the teeth and also by systemic factors related to bone metabolism. Some drugs used by patients during orthodontic treatment can also influence these tissue changes. The combined effect of mechanical forces along with the action of the drugs can be additive, inhibiting or synergistic [15,16].

Evaluation of the effect of LC on bone has been carried out in humans and in rats and has shown conflicting results such as inhibiting bone formation [17], no influence on bone [18], increased bone formation [19–21], or bone loss [22]. Baran et al [17] found that lithium treatment inhibits osteoid synthesis, leading to a decrease in bone mineralization in rats. However, Cohen et al [18] did not detect any effect on bone density after either short- or long-term treatment with lithium carbonate. On the other hand, the Clement-Lacroix et al [19] experimental study showed an anabolic effect of lithium on bone mass in rats.

In the context of orthodontia, Tang et al [21] found that the administration of a daily dose of Lithium in 4 week-old rats, administered by gavage of 200 mg/kg for 3, 7 and 14 days, increases new bone formation in the median palatine suture, despite having been initially delayed on day 3 of the rats subjected to rapid maxillary expansion. Only one study, Wang et al [23], evaluated the effect of Lithium chloride during induced tooth movement in rats. They verified that the average distance measured in the experimental group was slightly less than in the control group, with no statistically significant differences. However, these authors investigated the effects of lithium administered in acute form for 14 days, every 48 h. To simulate the use with patients, it is necessary evaluate chronic use of this drug. However, there are no studies that evaluate the effect of chronic use of LC on induced tooth movement.

Taking into consideration the controversial results found in the literature and the possible influence of LC on induced tooth movement, this study aimed to evaluate induced tooth movement in rats subjected to chronic use of lithium carbonate.

## Material and Methods

### Ethics statement

The name of the Institutional Animal Care and Use Committee that approved this study is the Ethics Committee on the Use of Animals of the Pontifícia Universidade Católica do Paraná, and the associated permit number is 631/11.

### Animals

The sample was composed of 192 male Wistar rats (*Rattus norvegicus albinus*), 9 weeks old, weighing about 300–350 g. The animals were husbanded in cages that were cleaned daily, respected the light/dark cycle, had a controlled temperature of 25±1°C, and were monitored daily. The rats’ diet was *ad libitum* and consisted of ration and water. All efforts were made to minimize suffering and distress, sedation was performed using Tiletamina/Zolazepan for the orthodontic device installation, and euthanasia was performed using an overdose of anesthetic solution.

The animals were randomly placed in 3 groups.

- L Group (n = 64), received a daily intraperitoneal administration of 60mg/kg of LC in saline solution (*Bioarte Farmácia de Manipulação Ltda*., Piracicaba, SP, Brazil), a dose that resulted in lithium serum levels of 1.30 ± 0.55 mmol/L, 90 minutes after the last administration[16]. The purity and power of the drug were certified by the manufacturer. The administration was performed using U-100 Insulin ½ cc 0.05 m (BD ultra-fine^TM^ BD- Becton Driver, Franklin Lakes) disposable plastic syringes. These animals were not subjected to induced tooth movement and were euthanized after 33, 37, 44 and 51 days (n = 16 in each period) of application of the drug. The therapeutic levels of 0.8 to 1.5 mmol/L is recommended for humans and also used in rats [22]. A previous study considered the acute use of lithium to be equivalent to a single dose, and chronic use to be equivalent to daily administration of the drug for three weeks [24]. In the present study, lithium was administered to the rats for more than three weeks (33, 37, 44 and 51 days), therefore characterizing chronic use.
- LM group (n = 64), received prior daily administration of LC as described above, resulting in serum lithium levels of 1.34 ± 0.53 mmol/L. The drug was administered for 30 days and during the subsequent 3, 7, 14 and 21 days, corresponding to the period of induced tooth movement [25]. The animals were euthanized on days 33, 37, 44 and 51.
- SM group (n = 64) received administrations of saline solution (*LBS-Laborasa Indústria Farmacêutica LTDA*, São Paulo, SP, Brazil), in volumes and periods equivalent to those used for the previous groups. Tooth movement and euthanasia were carried out in accordance with the aforementioned periods.

The animals were evaluated for the possible presence of diarrhea, polyuria and polydipsia. The rats were weighed at the beginning (ip) and at the end (fp) of the experimental time using an electronic precision balance (Gehaka-BG 4001, São Paulo, SP, Brazil). The actual body weight was calculated as a percentage, using the formula:

Actual body weight (%) = (ip/fp−1)x100

### Experimental protocol

**Fig 1:**
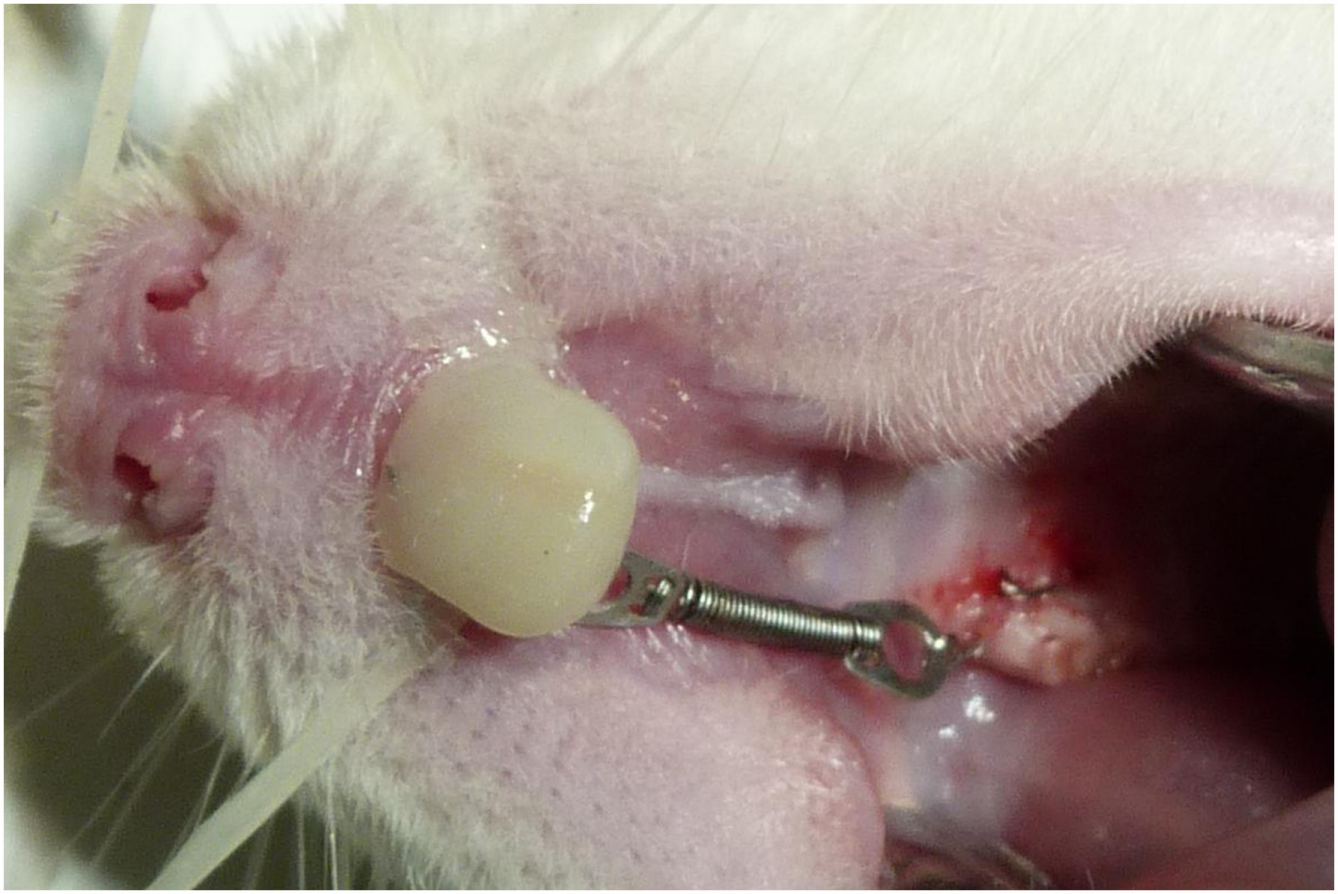
Orthodontic device installed.

The animals were sedated with an intramuscular injection of 50 mg/kg of Tiletamina/Zolazepan (Zoletil^®^ 50, *Brasil indústria e comércio Ltda*, Jurubatuba, São Paulo, SP, Brazil), for the induced tooth movement. The orthodontic device consisted of a closed, nickel titanium spring (GH^®^ Wire Orthodontics, Franklin) and stainless steel tying wire (Dental Morelli Ltda, São Paulo, SP, Brazil) of 0.025 mm, to attach the spring to the first upper right molar and to the upper incisor on the same side. That produced a 30cN [26–28] reciprocal force on the distal face of incisor and on the mesial face of the molar, measured using a dynamometer (Haag-Streit AG, Koeniz, Switzerland). The end of the tying wire was attached to the upper right incisor, using composite resin (4 seasons, Ivoclar Vivadent, Schaan, Liechtenstein, Luxembourg) after being conditioned with 37% phosphoric acid (Total Etch, Ivoclar Vivadent, Schaan, Luxembourg) and an application of light-cured adhesive (AdheSE, Ivoclar Vivadent, Schaan, Liechtenstein, Luxembourg) (Fig 1).

### Measurement of tooth movement

The distance between the first molar and the central incisor, prior to placement of the orthodontic device (initial measurement—IM) and after euthanasia (final measurement—FM), was measured using a digital from the most cervical point of the upper right incisor and the most cervical point of the 1st right molar. In these locations, there was no fixing resin of the orthodontic device, ensuring standardization of the reference points (Absolute-Mitutoyo, Kawasaki-Shi, Japan). The actual measurement of tooth movement was calculated using the formula:

The actual measurement of tooth movement (%) = (IM/FM−1)×100

### Biochemical analysis

The animals were euthanized using an overdose of anesthetic solution (100 mg/kg) of sodium pentobarbital (Syntec, Cotia, SP, Brazil), administered intraperitoneally, and all efforts were made to minimize suffering.

Blood samples from the animals were collected by intracardiac puncture at the moment of euthanasia. 6 ml of blood were collected in disposable vacutainer tubes with gel (BD-Becton Driver, Franklin Lakes, New Jersey), without anticoagulant.

Biochemical blood analysis was performed in the Lanac clinical analysis laboratory (Curitiba, PR, Brazil), and the following were quantified: the plasma level of inorganic phosphate (PO_4_), alkaline phosphatase (ALP), aspartate aminotransferase (AST), creatinine and calcium, using the Advia 1200/1800 (Medcorp-Siemens, Brasília, DF, Brazil) automatic biochemistry system. The serum level of lithium was measured using the Dimension RXL (Medcorp-Siemens, Brasília, DF, Brazil) automatic analyzer; the serum level of albumin was measured using the Advia 1200 (Medcorp-Siemens, Brasília, DF, Brazil) automatic biochemistry analyzer. The corrected total serum calcium values (alb-adjCa) obtained were adjusted for albumin and calculated using the formula:

alb-adjCa = [(40−albumin)×0.025 + total calcium]

[29]

### Histopathological analysis

Following euthanasia, the maxilla of every animal was removed, dissected and sectioned on the mid-line. The right hemi-maxilla was set in a 10% formalin solution for 24 hours and demineralized with 5% EDTA for 2 months.

After demineralization, the specimens were processed and embedded in paraffin in the Experimental Pathology Laboratory of PUCPR. Five cross sections, 4 μm thick, were obtained from the cervical third of the mesio-buccal root of the first molar, cut using a microtome with the occlusal surface of the molar parallel to the microtome and a 60 μm interval between each section. These sections were stained using the TRAP histochemistry technique. The TRAP enzyme is considered a specific marker for osteoclasts and can be used to determine bone resorption quantitatively [30]. TRAP staining was performed using the TRAP kit 387 (Sigma-Aldrich Co, St Louis, MO) according to the manufacturer's instructions.

In each section, 5 images of the mesial region of the mesio-buccal root of the first molar were captured, using a BX-50 Olympus microscope (Olympus, Tokyo, Japan) attached to a Dinolite^®^ AM 423X micro-camera (AmMo Electronics Corporation, New Taipei City 241, Taiwan) at 400X magnification [14]. The image acquisition parameters were set during the capture process. The number of osteoclasts was counted using the Image Pro-Plus 4.5 morphometry program (Media Cybernetics, Silver Spring, MD), with which a count grid was created. The multinucleated, TRAP-positive cells located in the periodontal ligament adjacent to the alveolar bone were considered to be osteoclasts. The number of osteoclasts was obtained by calculating the mean of the five sections [14].

### Statistical analysis

The SPSS 19.0 program was used for the statistical analysis (SPSS Inc, Chicago, Illinois). The Kolmogorov-Smirnov test of normality showed that the variables displacement rate, weight variation, corrected total calcium, PO_4_, ALP and creatinine presented normal distribution. Despite the fact that the variables AST and number of osteoclasts did not show normal distribution, the mean and median values were close, indicating symmetrical distribution. The two-way ANOVA, full factorial design was performed for the variables above. Since serum Lithium is a constant for the SM group at all times, the non-parametric Kruskal-Wallis test was used. Levene's Test for Homogeneity of Variance showed that the variables displacement rate, corrected total calcium, serum PO_4_, ALP, creatinine, AST and number of osteoclasts presented heterogeneous variances; therefore, the parametric Games-Howell multiple comparisons test was used. For serum Lithium, the non-parametric Dunn’s multiple comparisons test was used. The significance level adopted for all tests was 5% (p<0.05). The reproducibility power of the count of osteoclasts was assessed and it was observed that the Dahlberg error was 0.037%, indicating that the single evaluator reproduced the measurements reliably [31,32].

## Results and Discussion

The LM group showed a lower rate of movement at 44 days, when compared with the SM group (Table 1).

**Table 1:** Rate of induced tooth movement (%) in the LM group compared with the SM group, on days 33, 37, 44 and 51.

Group/ days	SM (Mean ±SD)	LM (Mean ±SD)
33 days	4.05 ± 3.61 A	1.76 ± 1.29 A
37 days	5.23 ± 3.23 A	3.02 ± 2.00 A
44 days	6.22 ± 3.90 A	1.84 ± 1.49 B
51 days	6.13 ± 3.53 A	3.14 ± 2.10 A

Two-way ANOVA, full factorial design, p> 0.05; Games Howell Test: different letters in the same line indicate statistically significant differences.

A higher serum lithium level was observed in the L and LM groups. In the SM group, a higher level of serum PO_4_ was observed. In addition, the LM group showed a higher mean value than the L group. A higher value for ALP was verified in the L group when compared with the SM and LM groups.

The creatinine variable showed a lower level in the LM group when compared with the L and SM groups. Weight variation was higher in animals from the L and LM groups (Table 2).

**Table 2:** Means and standard deviations of the variables: serum lithium, corrected total calcium, serum PO_4_, aspartate transaminase (AST), serum alkaline phosphatase (ALP), serum creatinine and weight variation in the groups Saline Solution and movement (SM), Lithium (L), and Lithium and Movement (LM).

	Mean ±SD		
Group/ Variables	SM	L	LM
**Intact serum lithium (mmol/L)**^1^^a^	0.10±0.00 A	1.30± 0.55 B	1.34±0.53 B
**Corrected total Ca (mg/dL)**^2^^b^	9.92±0.40 A	10.06±0.33 A	9.96±0.43 A
**Serum PO**_**4**_ **(mg/dL)**^2^^b^	9.94±1.92 A	7.59±1.17 B	7.08±1.48 B
**Serum AST (U/L)**^2^^b^	155.16±42.02 A	180.71±48.26 B	204.21±47.70 C
**Serum ALP (U/L)**^2^^b^	120.56±43.22 A	154.63±56.44 B	127.17±45.08 AB
**Serum creatinine (mg/dL)**^2^^b^	0.34±0.08 A	0.31±0.05 A	0.27±0.05 B
**Weight variation (%)**^2^^b^	8.01±7.24 A	23.34±11.59 B	24.55±12.37 B

^1^ Kruskall-Wallis Test, p<0.05;

^2^ two-way ANOVA full factorial design, p<0.05;

^a^ Dunn’s Test;

^b^ Games-Howell’s test;

Different letters in the same line indicate statistically significant differences.

**Fig 2:**
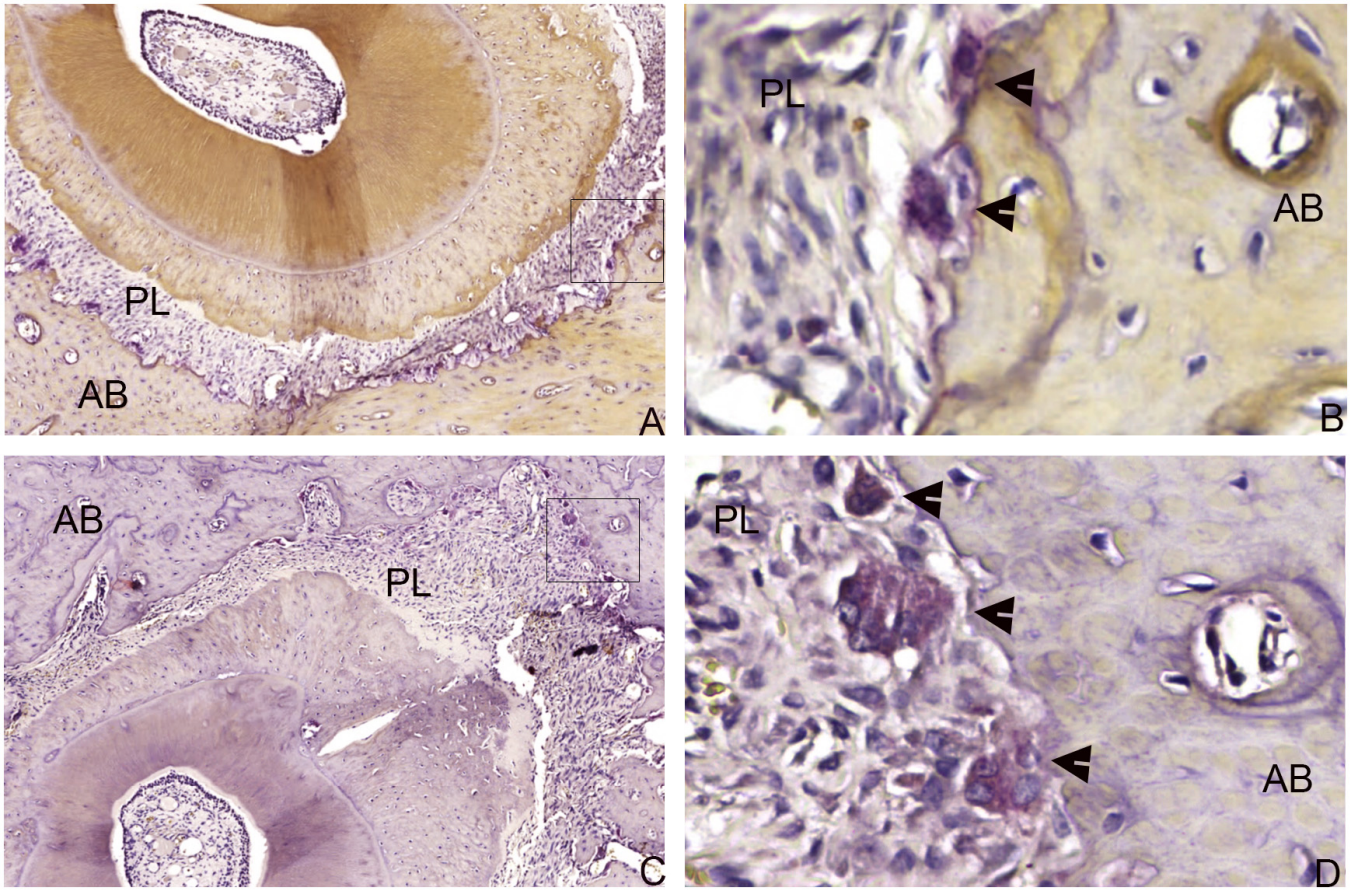
A tendency toward reduction in the number of osteoclasts was observed in the LM group (A, B) compared with the SM group (C, D) at 44 days. Osteoclasts (black arrows)—Multinucleated, TRAP-positive cells located in the periodontal ligament (PL) adjacent to the alveolar bone (AB) (TRAP, A, C 200X, B, D 400X magnification).

The number of osteoclasts in the L group remained constant at all times. No statistically significant differences were observed between the SM and LM groups at any times, although a tendency toward reduction in the number of osteoclasts in the LM group at 44 days (Table 3, Fig 2) was observed.

**Table 3:** Means and standard deviations of the variable number of osteoclasts in the groups lithium and movement (LM), saline solution and movement (SM) and lithium (L) on days 33, 37, 44 and 51.

Group / days	SM (Mean ±SD)	L (Mean ±SD)	LM (Mean ±SD)
33 days	1.54±0.93 A	0.23±0.25 B	1.13±0.48 A
37 days	0.93±0.95 A	0.21±0.31 A	0.61±0.40 A
44 days	2.56±1.26 A	0.17±0.30 B	1.60±1.32 A
51 days	1.18±0.96 A	0.16±.28 B	0.96±0.98 AB

Two-way ANOVA full factorial design, p<0.05; Games-Howell’s test: Different letters in the same line indicate statistically significant differences.

Lethargy, diarrhea, increased urination and water intake were clinically observed in animals subjected to administration of LC, as compared to the SM group.

This experimental study assessed induced tooth movement in rats subjected to the chronic use of lithium carbonate by daily administration of LC within the therapeutic levels (0.8 to 1.5 mmol/L) recommended for both humans and rats [22]. The serum LC levels reached in this study were 1.30 ± 0.55 mmol/L for the L group and 1.34 ± 0.53 mmol/L for the LM group.

This is the first study to identify a lower rate of tooth displacement in rats subjected to chronic use of LC during the 14 days of induced tooth movement. These 14 days correspond to the animals that received lithium for 30 days and then for the 14 days following induced tooth movement; i.e., euthanized on the 44^th^ day. A possible explanation for this fact is the reduction in the number of osteoclasts during this period which, although not statistically significant, may have caused less bone resorption resulting in the lower rate of tooth movement clinically observed. In a previous study, Spencer et al. [33] verified *in vitro* that lithium chloride (LiCl) inhibits osteoclastogenesis in co-cultures of rat osteoblasts and spleen mononuclear cells. In addition, in a previous study, it was found that lithium induces apoptosis in macrophages [34]. Osteoclasts are derived from granulocyte-macrophage colony forming units in bone marrow, cells then fuse and differentiate into mature osteoclasts [35]. However, there are no studies that evaluate the lithium-induced apoptosis in osteoclasts. Further *in vitro* studies may clarify this possible effect of lithium on osteoclasts. The number of osteoclasts remained constant in Group L, as was expected, because there was no induced tooth movement in this group.

Lithium is a well-documented gsk-3 inhibitor and can activate the wnt/beta-catenin pathway. Wnt ligands are local signaling factors involved in bone remodeling and the pharmacological modulation of this pathway may influence bone mass. Activation of the Wnt signaling pathway may be done with lithium [19, 36, 37]. The wnt/beta-catenin pathway increases bone mass through the stem cell stimulus of preosteoblast multiplication and osteogenesis, and also blocks the osteoblasts and osteocyte apoptose. In addition, by improving the osteoprotegerin/ receptor activator of the NF-kB ligand ratio, β-catenin inhibits osteoclastogenesis [38]. Future studies must be conducted to evaluate this signaling pathway during induced tooth movement in rats submitted to chronic use of LC.

Within 21 days, the rate of movement was observed to be similar between Groups LM and SM; however, this observation was verified in just one, single moment of activation. Further studies should be conducted in order to determine whether, after reactivation of the orthodontic devices, the reduction in the tooth movement observed within 14 days can be cumulative and represent an important delay in induced tooth movement. This would make individualization in both the diagnosis and the orthodontic treatment plan necessary. In the present study, tooth movement in the LM group was observed to be less than in the SM group at the 44^th^ day of administration of lithium. This corresponded to 14 days of induced tooth movement. This difference was also observed by Wang et al. [23]; however, with no statistically significant differences. It is possible that these authors did not find any differences due details in the methodology, such as time (acute form—14 days, every 48 h during the experiment) and method (gavage-fed) of administration of the drug. In addition, the method of gavage-fed administration may result in variable blood levels of lithium; however, these authors did not perform this measurement. In addition, only the induced tooth movement and root resorption area were evaluated.

Every time a drug is administered over a long period of time, there is the risk of the organism adapting to it, producing an effect of drug tolerance [39]. The fact that the drug produces less effect on the 44th day compared to the 37th can be explained by the fact that the body is not responding as well to the drug and is presenting a chronic and tolerant effect to the medication.

Biochemical studies may be useful in assessing induced tooth movement [9, 10, 12]. The significant increase of calcium and PO_4_ is considered a result of bone demineralization [40]. However, in the present study, no change was found in the calcium and there was a decrease of PO_4_ in the rats subjected to chronic use of LC, which does not indicate bone demineralization. PO_4_ is critical for many physiological functions, including skeleton development and mineral metabolism, among others [40]. This reduction in PO_4_ levels could be related to chronic diarrhea [41]. Diarrhea, polydipsia and polyuria have been reported in association with the use of this drug [42]. These results were also found in this study. This decrease in PO_4_ was not observed in some studies [17, 22] in which the levels did not change with the administration of LC, and are contrary to the results observed by Sharman et al [43] in which the serum level increased. These differences may be related to differences in methodology, such as: studies in animals and humans, gender, induced tooth movement, time and dose of treatment with the LC, and the form of administration.

ALP is a systemic marker for bone formation and has been used to assess bone formation during induced tooth movement [9, 10, 44]. In this study, a higher ALP value was observed in the L group when compared with the LM group. This suggests that induced tooth movement reduces ALP in patients using LC. Likewise, Milne et al [9] also found a decrease in ALP levels after applying orthodontic forces, consistent with histologically observed reduction in bone mass. During induced tooth movement, resorption occurs on the compression side and new bone formation on the traction side [8]. This bone mass reduction, verified against the application of orthodontic forces, may be related to bone resorption. The effect of lithium carbonate on bone and its biology has not yet been completely elucidated [20]. In this study, when the animals were subjected to LC and induced tooth movement, ALP levels similar to those of the rats in the SM group were observed, not changing, in this case, bone deposition.

A quantitative analysis of creatinine serum levels may indicate kidney damage [45]. In the present study, creatinine levels below the maximum normal limit (0.65 mg/dL) [46] for Wistar rats were found in all groups, indicating absence of kidney damage. Differing from the present study, Allagui et al [47] found significant increase in the creatinine levels in the animals of the group treated with LC. This indicates kidney lesions that may be associated with the high dose of the drug administered in the food in order to reach the therapeutic serum levels. Close et al [48]. verified that LC is related to a higher chance of kidney failure. Kidney lesions are frequently observed in patients subjected to lithium therapy for more than one year [47]. In the present study, LC was administered for 51 days, the longest period of therapy.

The animals treated with LC showed greater weight gain, in agreement with the results of Mcknight et al [49], in which the association between lithium and weight gain may be due to the property, similar to that of insulin, of increasing the cellular uptake of glucose, increasing thirst, and direct stimulation of the appetite center [49].

A lower rate of tooth movement after 14 days and no alteration after 21 days were verified with the use of Lithium Carbonate. It was also observed that Lithium Carbonate did not change bone deposition when associated with induced tooth movement indicating that this drug, combined with induced tooth movement, does not appear to interfere in bone deposition.

Human studies, regarding liver and kidney cytotoxicity, are necessary in order to monitor patients who use lithium carbonate and are subjected to orthodontic treatment during the entire orthodontic treatment period.

Lithium Carbonate provides a slower rate of absorption, which may indicate a greater therapeutic effect, lower solubility and lower molecular weight when compared with lithium chloride or sulphate [50].

According to the findings of the present study, multidisciplinary interaction among the orthodontist, psychiatrist and general physician is mandatory in the approach to the treatment of these patients [51, 52].

## Conclusions

Induced tooth movement associated with chronic use of lithium carbonate in Wistar rats results in:

- Lower rate of tooth movement during a 14-day period, possibly due to the reduction in the number of osteoclasts.
